# Index admission cholecystectomy for biliary acute pancreatitis or choledocholithiasis reduces 30-day readmission rates in children

**DOI:** 10.1007/s00464-024-10790-2

**Published:** 2024-03-19

**Authors:** Sagar J. Pathak, Patrick Avila, Sun-Chuan Dai, Mustafa A. Arain, Emily R. Perito, Abdul Kouanda

**Affiliations:** 1 Division of Pediatric Gastroenterology, Department of Pediatrics, University of California, San Francisco, San Francisco, CA USA; 2 Division of Gastroenterology, Department of Medicine, University of California, San Francisco, San Francisco, CA USA; 3 Division of Gastroenterology, Department of Medicine, Center for Interventional Endoscopy, AdventHealth, Orlando, FL USA

**Keywords:** Pediatric, Pancreatitis, Hepatobiliary disorders, Surgery, Endoscopic retrograde cholangiopancreatography (ERCP), Equity

## Abstract

**Background:**

Adult patients with biliary acute pancreatitis (BAP) or choledocholithiasis who do not undergo cholecystectomy on index admission have worse outcomes. Given the paucity of data on the impact of cholecystectomy during index hospitalization in children, we examined readmission rates among pediatric patients with BAP or choledocholithiasis who underwent index cholecystectomy versus those who did not.

**Methods:**

Retrospective study of children (< 18 years old) admitted with BAP, without infection or necrosis (ICD-10 K85.10), or choledocholithiasis (K80.3x–K80.7x) using the 2018 National Readmission Database (NRD). Exclusion criteria were necrotizing pancreatitis with or without infected necrosis and death during index admission. Multivariable logistic regression was performed to identify factors associated with 30-day readmission.

**Results:**

In 2018, 1122 children were admitted for index BAP (*n* = 377, 33.6%) or choledocholithiasis (*n* = 745, 66.4%). Mean age at admission was 13 (SD 4.2) years; most patients were female (*n* = 792, 70.6%). Index cholecystectomy was performed in 663 (59.1%) of cases. Thirty-day readmission rate was 10.9% in patients who underwent cholecystectomy during that index admission and 48.8% in those who did not (*p* < 0.001). In multivariable analysis, patients who underwent index cholecystectomy had lower odds of 30-day readmission than those who did not (OR 0.16, 95% CI 0.11–0.24, *p* < 0.001).

**Conclusions:**

Index cholecystectomy was performed in only 59% of pediatric patients admitted with BAP or choledocholithiasis but was associated with 84% decreased odds of readmission within 30 days. Current guidelines should be updated to reflect these findings, and future studies should evaluate barriers to index cholecystectomy.

**Supplementary Information:**

The online version contains supplementary material available at 10.1007/s00464-024-10790-2.

Gallstones are the most frequent cause of acute pancreatitis among adults worldwide, and though pediatric gallstone disease was previously considered rare, it is an increasingly recognized condition in children, with prevalence drastically increasing over time [1–5]. Risk factors in children include obesity, rapid weight loss, hemolytic disorders, cystic fibrosis, and inflammatory bowel disease [3].

The benefits of early cholecystectomy for biliary acute pancreatitis (BAP) and choledocholithiasis have been well established and include lower readmission, lower hospital length of stay, and less biliary-related complications [6–8]. However, recommendations for management of pediatric gallstone disease is largely based on small population studies, single center studies, or expert opinion [9–11]. The most recent pediatric pancreatitis guidelines on the management of acute pancreatitis in the pediatric population highlight the importance of considering early cholecystectomy for biliary pancreatitis, though the current pediatric data underlying these recommendations is limited: a foundational, though small, cohort of 19 patients showing no adverse events for early cholecystectomy, and recurrence of pancreatitis for those who had delayed surgery [11, 12]. More recently, a multicenter cohort retrospective analysis similarly showed a higher recurrence in disease for those with delayed cholecystectomy [13]. However, there remain no data on national rates of cholecystectomy among those with mild biliary acute pancreatitis or choledocholithiasis nor of subsequent 30-day readmission.

Early recognition and treatment of pediatric gallstone disease may prevent the development of complications and improve the long-term outcomes for affected children. Therefore, we aimed to evaluate national level practice of cholecystectomy during index admission in the US and to identify predictors of 30-day readmission using the National Readmission Database (NRD) among the pediatric population.

## Materials and methods

### Data source and study design

This is a retrospective cohort study of admissions to US acute-care hospitals for biliary acute pancreatitis and choledocholithiasis among children and young adults. Data on hospital admissions of all pediatric patients (age < 18 years) in 2018 was extracted from the National Readmissions Database (NRD). NRD is an inpatient database with several key features: it provides sufficient data for analysis across hospital types and the study of readmissions for relatively uncommon disorders and procedures, contains discharge data from 27 geographically dispersed states, accounting for 57.8% of the total US resident population and 56.6% of all US hospitalizations, is designed to support national readmission analyses. Of note, this database links patient readmission to the same or any other hospital in the USA for each calendar year (1 January through 31 December) but does not link patient data across preceding or subsequent years. Therefore, we excluded index admissions occurring in December from our analysis since readmissions for those encounters could not be tracked. The database excludes observation admissions, rehabilitation hospitals, and chemical-dependency units. This study was exempted from IRB review, as no identifiable patient data were included in the database.

### Study population

We used *International Classification of Diseases, Tenth Revision, Clinical Modification* (ICD-10-CM) codes to identify all hospitalized pediatric patients (age < 18 years) with a primary diagnosis of mild biliary acute pancreatitis (ICD-10 K85.10) or choledocholithiasis (K80.3x–K80.7x) who survived to hospital discharge. Both cholecystectomy and ERCP procedures performed were identified using ICD procedure codes. All diagnostic and procedural codes used for classifications are shown in Supplementary Table 1. Admissions were identified by querying for all diagnostic ICD-10-CM codes corresponding to biliary acute pancreatitis and choledocholithiasis. We excluded patients with biliary acute pancreatitis with features of necrosis or infection to characterize those with mild pancreatitis and to address bias introduced by those with severe disease. We also excluded patients who were coded as having elective readmission.

### Definitions of variables

The NRD collects demographic information, including age, sex, income, and primary and secondary insurance as well as hospital information (e.g. bed size, location, and teaching status). The All Patients Refined Diagnosis Related Groups (APR-DRG) severity score is was used to assess symptom severity, which has bene shown to be accurate in the pediatric population [14, 15]. The NRD definition of hospital size is available in Supplementary Table 2.

### Outcomes

The primary outcome of this study was readmission within 30 days after discharge date in patients admitted with mild biliary acute pancreatitis or choledocholithiasis to US hospitals. The primary predictor was index or delayed admission cholecystectomy. We performed a multivariable regression analysis to identify predictors of all-cause 30-day readmission in these patients. We additionally evaluated a secondary predictor of ERCP during index admission in patients admitted with choledocholithiasis to US hospitals. Multivariable regression analysis was performed for this limited cohort using conserved predictors of initial model.

### Statistical analysis

Data are presented as raw number (*n*) and weight frequency (%) for categorical variables or mean and standard deviation (SD) for continuous variables. Univariate analysis was first performed to assess differences between the two groups (no cholecystectomy during index admission vs cholecystectomy during index admission); categorical variables were compared by using *χ*^2^ tests and continuous variables using *t* tests. Univariate logistic analysis was performed and to identify significant predictors and factors with *p* < 0.10 were considered in multivariable logistic regression—adjusting for patient and hospital characteristics. Backward selection was employed using *p* < 0.05 for retention in the final model. Subgroup analysis was conducted on the choledocholithiasis—only group to evaluate readmission rates based on ERCP utilization. The final model for subgroup analysis conserved significant predictors of the initial model. Results from the multivariable analyses were represented using odds ratios (OR) and 95% confidence intervals. Model performance was confirmed with both Pearson and Hosmer–Lemeshow goodness-of-fit testing. All analyses were performed with STATA (Version 17, College Station, TX).

## Results

### Baseline patient characteristics

We identified 1122 unique inpatient hospitalizations with either a diagnosis of mild biliary acute pancreatitis or choledocholithiasis [377 (33.6%) and 745 (66.4%), respectively]. (Table 1) Patients who underwent cholecystectomy were more likely to be female (76.9% vs 61.4%, *p* < 0.001) and older in age (14.5 vs 12.0, *p* < 0.001). Those with length of stay less than 7 days was also associated with cholecystectomy performance (81.5% vs 53.8%, *p* < 0.001). Overall, only 59% of patients underwent cholecystectomy during the index admission. Among all 1,122 children, 296 (26.4%) were readmitted within 30 days. Those who underwent cholecystectomy had a significantly lower prevalence of readmission than those who did not, for both biliary pancreatitis (5.4% vs 20.7%, *p* < 0.001) and choledocholithiasis (14.4% vs 58.3%, *p* < 0.001).

**Table 1:** Baseline characteristics of patients with biliary acute pancreatitis or choledocholithiasis by cholecystectomy status

	Overall (*n* = 1122)	Stratified by cholecystectomy status		
	*n*, % mean, SD	No cholecystectomy (*n* = 459)	Cholecystectomy (*n* = 663)	*p* value
Sex				< 0.001
Female	792 (70.6%)	282 (61.4%)	510 (76.9%)	
Age	13.5 (4.2)	12.0 (5.2)	14.5 (2.9)	< 0.001
Weekend Admission	262 (23.4%)	111 (24.2%)	151 (22.8)	0.58
Hospital size				0.36
Small	168 (15.0%)	67 (14.6%)	101 (15.2%)	
Medium	158 (14.1%)	57 (12.4%)	101 (15.2%)	
Large	796 (70.9%)	335 (73.0%)	461 (69.5%)	
Hospital type				0.46
Government	188 (16.8%)	75 (16.3%)	113 (17.0%)	
Private, not-for-profit	864 (77.0%)	360 (78.4%)	504 (76.0%)	
Private, profit	70 (6.2%)	24 (5.2%)	46 (6.9%)	
Hospital location and teaching status				< 0.001
Metropolitan non-teaching	84 (7.5%)	22 (4.8%)	62 (9.4%)	
Metropolitan teaching	1,006 (89.7%)	430 (93.7%)	576 (86.9%)	
Nonmetropolitan hospital	32 (2.9%)	7 (1.5%)	25 (3.8%)	
Length of stay				< 0.001
< 7 days	787 (70.1%)	247 (53.81%)	540 (81.5%)	
≥ 7 days	335 (29.9%)	212 (46.2%)	123 (18.6%)	
Payer				0.91
Medicare	8 (0.71%)	4 (0.9%)	4 (0.6%)	
Medicaid	708 (63.1%)	286 (62.3%)	422 (63.7%)	
Private insurance	353 (31.5%)	148 (32.2%)	205 (30.9%)	
Self-pay/other	53 (4.7%)	21 (4.6%)	32 (4.8%)	
Median household income				0.67
0–25th percentile	370 (33.5%)	151 (33.5%)	219 (33.5%)	
26–50th percentile	350 (31.7%)	145 (32.2%)	205 (31.4%)	
51–75th percentile	220 (19.9%)	83 (18.4%)	137 (21.0%)	
76–100th percentile	164 (14.9%)	72 (16.0%)	92 (14.1%)	
ERCP performed	363 (32.4%)	114 (24.8%)	249 (37.6%)	< 0.001
Biliary pancreatitis	76 (20.9%)	–	–	
Choledocholithiasis	287 (79.1%)	–	–	
Diagnosis				< 0.001
Biliary pancreatitis	377 (33.6%)	116 (25.3%)	261 (39.4%)	
Choledocholithiasis	745 (66.4%)	343 (74.7%)	402 (60.6%)	
APR-DRG category				< 0.001
Minor	218 (19.4%)	96 (20.9%)	122 (18.4%)	
Moderate	433 (38.6%)	168 (36.6%)	265 (40.0%)	
Major	395 (35.2%)	133 (29.0%)	262 (39.5%)	
Extreme	76 (6.8%)	62 (13.5%)	14 (2.1%)	
Readmission rate (overall)	296 (26.4%)	224 (48.8%)	72 (10.9%)	< 0.001
Biliary pancreatitis	38 (10.1%)	20.7%	5.4%	
Choledocholithiasis	258 (34.6%)	58.3%	14.4%	

Data are presented as mean (SD) for continuous measures, and n (%) for categorical measures

### Predictors of 30-day all-cause readmissions: entire cohort

We performed univariate and subsequent multivariable logistic regression analysis to identify predictors of 30-day all-cause readmission following the index admission for patients admitted with BAP or choledocholithiasis.

In univariate analysis, female sex (OR 0.54, CI 0.41–0.72, *p* < 0.001), age (OR 0.86, CI 0.84–0.90, *p* < 0.001), admission to a private for-profit hospital (OR 0.36, CI 0.17–0.77, *p* = 0.008), private insurance (OR 0.70, CI 0.52–0.95, *p* = 0.020), median house hold income in the 26-50th percentile (OR 0.69, CI 0.50–0.96, *p* = 0.027), ERCP performed during index admission (OR 0.22, CI 0.15–0.32, *p* < 0.001), and undergoing index cholecystectomy were all associated with decreased odds of 30-day readmissions. Length of stay ≥ 7 days (OR 20.04, CI 14.37–27.94, *p* < 0.001) and APR-DRG category of “extreme” (OR 3.47, CI 2.01–5.99, *p* < 0.001) were associated with an increased odds of 30-day readmission.

In the multivariable model, an APR-DRG severity score of “extreme” (OR 0.28, 95% CI 0.13–0.59) and undergoing index cholecystectomy (OR 0.16, CI 0.11–0.24, *p* < 0.001) were associated with decreased odds of 30-day readmissions. Patients with ≥ 7 days length of stay had higher likelihood of readmission (OR 21.45, 95% CI 14.14–32.52).

### Predictors of 30-day all-cause readmissions: choledocholithiasis only

**Fig. 1:**
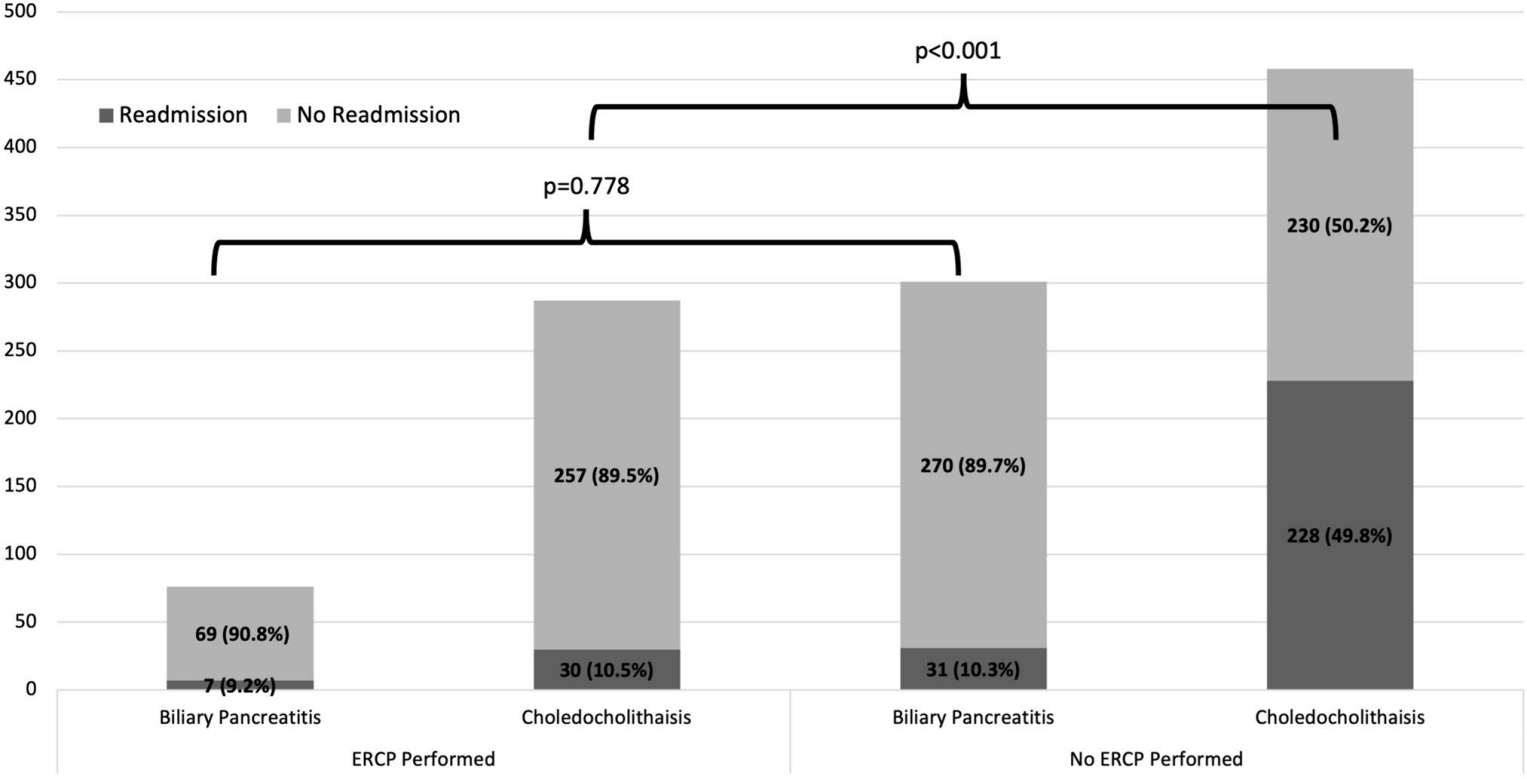
Readmission rate by ERCP status and diagnosis

In patients with choledocholithiasis, readmission rate was 49.8% if an ERCP was not performed compared to 10.5% if ERCP was performed (Fig. 1; Table 2). A preserved multivariable logistical regression model showed that ERCP for choledocholithiasis was associated with a 79% reduction in odds (OR 0.21, 95% CI 0.12–0.36) of readmission (Table 3). Additional covariates from the prior model (APR-DRG severity, stratified length of stay, and cholecystectomy) showed no significant differences (Table 3).

**Table 2:** Predictors of 30-day readmission for patients with biliary acute pancreatitis or choledocholithiasis

Predictors	Univariate regression		Multivariable logistic regression	
	Odds ratio (95% CI)	*p* value	Odds ratio (95% CI)	*p* value
Sex				
Male	Reference	Reference	Reference	Reference
Female	0.54 (0.41–0.72)	< 0.001	0.97 (0.93–1.02)	0.214
Age	0.86 (0.84–0.90)	< 0.001	1.02 (0.67–1.54)	0.925
Weekend admission				
No	Reference	Reference	*	*
Yes	0.79 (0.57–1.09)	0.145	*	*
Hospital size*				
Small	Reference	Reference	*	*
Medium	0.85 (0.51–1.42)	0.536	*	*
Large	1.19 (0.81–1.75)	0.374	*	*
Hospital type				
Government	Reference	Reference	Reference	Reference
Private, not-for-profit	0.89 (0.63–1.26)	0.503	**	**
Private, profit	0.36 (0.17–0.77)	0.008	**	**
Hospital location and teaching status				
Nonmetropolitan hospital	Reference	Reference	Reference	Reference
Metropolitan non-teaching	0.27 (0.07–1.08)	0.064	**	**
Metropolitan teaching	2.16 (0.82–5.65)	0.118	**	**
Length of stay				
< 7 days	Reference	Reference	Reference	Reference
≥ 7 days	20.04 (14.37–27.94)	< 0.001	21.45 (14.14–32.52)	< 0.001
Payer				
Medicaid	Reference	Reference	Reference	Reference
Medicare	0.35 (0.04–2.84)	0.325	**	**
Private insurance	0.70 (0.52–0.95)	0.020	**	**
Self-pay/other	0.57 (0.28–1.15)	0.115	**	**
Median household income				
0–25th percentile	Reference	Reference	Reference	Reference
26–50th percentile	0.69 (0.50–0.96)	0.027	**	**
51–75th percentile	0.70 (0.48–1.02)	0.070	**	**
76–100th percentile	0.76 (0.50–1.15)	0.201	**	**
APR-DRG category				
Minor	Reference	Reference	Reference	Reference
Moderate	1.03 (0.71–1.50)	0.861	1.10 (0.66–1.84)	0.709
Major	0.85 (0.58–1.25)	0.409	0.62 (0.36–1.08)	0.093
Extreme	3.47 (2.01–5.99)	< 0.001	0.28 (0.13–0.59)	0.001
ERCP performed				
No	Reference	Reference	Reference	Reference
Yes	0.22 (0.15–0.32)	< 0.001	0.28 (0.17–0.45)	< 0.001
Cholecystectomy performed				
No	N/A	N/A	Reference	Reference
Yes	0.13 (0.09–0.17)	< 0.001	0.16 (0.11–0.24)	< 0.001

Multivariable logistic regression model: threshold for entry into the multivariable model was *p* < 0.1 in univariate model. Threshold for retention in the final model was *p* < 0.05. Hospital size was determined by NRD criteria specified by hospital location and teaching status

*Not included in multivariable model

**Not retained in the final multivariable model

**Table 3:** Predictors of 30-day readmission for patients with choledocholithiasis only (*n* = 745)

Predictors	Multivariable logistic regression	
	Odds ratio (95% CI)	*p* value
Sex		
Male	Reference	Reference
Female	0.89 (0.63–1.25)	0.488
Age	0.98 (0.94–1.02)	0.561
Length of stay		
< 7 days	Reference	Reference
≥ 7 days	16.18 (11.11–23.57)	< 0.001
APR-DRG category		
Minor	Reference	Reference
Moderate	1.21 (0.66–2.21)	0.545
Major	0.62 (0.30–1.31)	0.212
Extreme	0.29 (0.11–0.78)	0.014
ERCP performed		
No	Reference	Reference
Yes	0.21 (0.12–0.36)	< 0.001
Cholecystectomy performed		
No	Reference	Reference
Yes	0.14 (0.08–0.24)	< 0.001

## Discussion

In the largest study to date on the impact of cholecystectomy on readmission rates in children with BAP or choledocholithiasis, we found that cholecystectomy performance was associated with lower prevalence of readmission and lower length of stay during index admission. In these children, the rationale for cholecystectomy during the index admission is to avoid the potential complications of delayed surgery, such as recurrent episodes of acute cholecystitis, pancreatitis, and cholangitis, which may require emergency surgery, longer hospital stays, and increased healthcare costs. This approach has been recommended in recent guidelines from the North American Society for Pediatric Gastroenterology, Hepatology, and Nutrition (NASPGHAN), but based on very limited data from smaller cohorts; this study provides data from a national cohort to support the recommendation [10–13, 16, 17].

Despite current recommendations, pediatric index cholecystectomy was only performed in 59% of admissions for biliary acute pancreatitis and choledocholithiasis. Interestingly, this trend is mirrored in multiple analogous retrospective studies in the adult literature, even after the foundational randomized controlled PONCHO trial, which showed that compared with delayed cholecystectomy, index admission cholecystectomy reduced rate of recurrent gallstone-related complications in patients with mild BAP [6–8, 18]. These findings were momentous in adult literature and emphasized the needless hospital readmission and healthcare utilization brought on by delayed cholecystectomy.

The adoption of index cholecystectomy likely varies among pediatric centers across the country. Several factors may influence the decision to perform index cholecystectomy in pediatric patients, including the severity of presentation and the availability of resources. However, given the burden of disease and healthcare utilization, the adoption of cholecystectomy on index admission in choledocholithiasis and mild BAP appears key to outcomes in children with gallstone disease. Though there is research reporting no difference in technical complexity between early and delayed cholecystectomy, there may remain concerns about performing surgery shortly after pancreatitis episode [19]. Additionally, though guidelines and recommendations represent ideal policies, their implementation may not always be pragmatic, particularly at hospitals with limited resources or those with economic constraints [20].

Although ERCP was performed in less than 40% of children with choledocholithiasis, it conferred a staggering 79% reduction in 30-day readmission when performed. This may reflect the limited number of pediatric advanced endoscopists and warrants further independent study to better characterize need and utilization of pediatric ERCP [21].

Our results showed that longer length of stay was associated with greater likelihood of readmission, though extreme severity of disease—as defined by APR-DRG—conveyed a lower likelihood of readmission after adjusting for significant factors. This was likely caused by the low number of patients with extreme severity of disease who had length of stay greater than 7 days allowing for greater skew of data.

We acknowledge several limitations to this study. This is a retrospective study, which can demonstrate associations but not establish causality. The use of a national database that is reliant on ICD-10 coding can include coding errors or omissions in the database. Additionally, the NRD does not have information on vitals, lab values, or imaging and thus disease severity to allow for granular analysis of children’s clinical conditions. Information on patients who received cholecystectomy as an outpatient after index admission was not obtainable, although we suspect this is rare. We also could not evaluate social risk factors for readmission. Nevertheless, this represents the largest study cohort in the pediatric population and suggests that cholecystectomy on index admission should be standard of practice for pediatric BAP and choledocholithiasis.

In conclusion, in children admitted for BAP or choledocholithiasis, hospital discharge before cholecystectomy was associated with a significantly increased odds of readmission. Further studies are needed to determine the barriers to cholecystectomy and differential availability based on patient and hospital-level factors. In addition, the development of standardized protocols could help to increase the adoption of pediatric index cholecystectomy and improve outcomes for children.

### Supplementary Information

Below is the link to the electronic supplementary material.

Supplementary file1 (DOCX 15 kb)
