# Supplementary material for: Index admission cholecystectomy for biliary acute pancreatitis or choledocholithiasis reduces 30-day readmission rates in children
Source: Surg Endosc. 2024 Mar 19;38(5):2649–56. doi: 10.1007/s00464-024-10790-2 (PMC11078817; doi:10.1007/s00464-024-10790-2)
Supplement: Supplementary file 1 — Supplementary file1 (DOCX 15 kb) [file 464_2024_10790_MOESM1_ESM.docx]

| Supplemental Table 1: Pancreatic and Biliary Disorder Diagnosis Codes (ICD-9 & 10) | |
| --- | --- |
| Diagnostic ICD Codes | **ICD-10 Codes** |
| Biliary Acute Pancreatitis | K85.10 |
| Choledocholithiasis | K80.30, K80.31, K80.32, K80.33, K80.34, K80.35, K80.36, K80.37, K80.40, K80.41, K80.42, K80.43, K80.44, K80.45, K80.46, K80.47, K80.50, K80.51, K80.60, K80.61, K80.62, K80.63, K80.64, K80.65, K80.66, K80.67, K80.70, K80.71 |
| Surgery Procedure Codes | **ICD-10 Codes** |
| Laproscopic cholecystectomy (open and laproscopic technique) | 0FT40ZZ, 0FT44ZZ |
| Endoscopic retrograde cholangiopancreatography | 0FJB8ZZ, 0FJD8ZZ, 0F9C8ZZ, 0F758DZ, 0F768DZ, 0F788DZ, 0F798DZ, 0F7C8DZ, 0F9580Z, 0F9680Z, 0F9880Z, 0F9980Z, 0F9C80Z, 0FC58ZZ, 0FC68ZZ, 0FC88ZZ, 0FC98ZZ, 0FCC8ZZ, 0FF58ZZ, 0FF68ZZ, 0FF88ZZ, 0FF98ZZ, 0FFC8ZZ, 0FJD8ZZ, 0F7D8DZ, 0F7F8DZ, 0F9D80Z, 0F9F80Z, 0FCD8ZZ, 0FCF8ZZ, 0FFD8ZZ, 0FFF8ZZ |

| **Supplemental Table 2: BEDSIZE CATEGORIES (Beginning in 1998)** | | | |
| --- | --- | --- | --- |
| Location and Teaching Status | **Hospital Bedsize** | | |
|  | Small | Medium | Large |
| **NORTHEAST REGION** | | | |
| Rural | 1-49 | 50-99 | 100+ |
| Urban, nonteaching | 1-124 | 125-199 | 200+ |
| Urban, teaching | 1-249 | 250-424 | 425+ |
| **MIDWEST REGION** | | | |
| Rural | 1-29 | 30-49 | 50+ |
| Urban, nonteaching | 1-74 | 75-174 | 175+ |
| Urban, teaching | 1-249 | 250-374 | 375+ |
| **SOUTHERN REGION** | | | |
| Rural | 1-39 | 40-74 | 75+ |
| Urban, nonteaching | 1-99 | 100-199 | 200+ |
| Urban, teaching | 1-249 | 250-449 | 450+ |
| **WESTERN REGION** | | | |
| Rural | 1-24 | 25-44 | 45+ |
| Urban, nonteaching | 1-99 | 100-174 | 175+ |
| Urban, teaching | 1-199 | 200-324 | 325+ |

HCUP NRD Description of Data Elements. Healthcare Cost and Utilization Project (HCUP). August 2015. Agency for Healthcare Research and Quality, Rockville, MD. www.hcup-us.ahrq.gov/db/vars/hosp_bedsize/nrdnote.jsp.
